# Angiotensin Converting Enzyme Activity in Alopecia Areata

**DOI:** 10.1155/2014/694148

**Published:** 2014-10-01

**Authors:** Mohammad Reza Namazi, Armaghan Ashraf, Farhad Handjani, Ebrahim Eftekhar, Amir Kalafi

**Affiliations:** ^1^Molecular Dermatology Research Center, Shiraz University of Medical Sciences, Shiraz 71348 44119, Iran; ^2^Department of Dermatology, Shiraz University of Medical Sciences, Shiraz 71348 44119, Iran; ^3^University of New South Wales, Sydney, NSW 2052, Australia; ^4^Molecular Medicine Research Center, Department of Biochemistry, Faculty of Medicine, Hormozgan University of Medical Sciences, Bandar Abbas 79149 64153, Iran

## Abstract

*Background*. Alopecia areata (AA) is a chronic inflammatory disease of the hair follicle. The exact pathogenesis of AA remains unknown, although recent studies support a T-cell mediated autoimmune process. On the other hand, some studies have proposed that the renin-angiotensin-aldosterone system (RAAS) may play a role in autoimmunity. Therefore, we assessed serum activity of angiotensin converting enzyme (ACE), a component of this system, in AA. *Methods*. ACE activity was measured in the sera of 19 patients with AA and 16 healthy control subjects. In addition, the relationship between severity and duration of the disease and ACE activity was evaluated. *Results*. Serum ACE activity was higher in the patient group (55.81 U/L) compared to the control group (46.41 U/L), but the difference was not statistically significant (*P* = 0.085). Also, there was no correlation between ACE activity and severity (*P* = 0.13) and duration of disease (*P* = 0.25) in the patient group. *Conclusion*. The increased serum ACE activity found in this study may demonstrate local involvement of the RAAS in the pathogenesis of AA. Assessment of ACE in a study with a larger sample size as well as in tissue samples is recommended in order to further evaluate the possible role of RAAS in AA.

## 1. Introduction

Alopecia areata (AA) is a nonscarring type of hair loss that results from chronic hair follicle inflammation. It presents as coin shaped patches of hair loss on normal skin (the most common form), especially on the scalp. Sometimes, the whole scalp is involved (alopecia totalis) and if the whole body is affected, it is called alopecia universalis [1]. AA probably has a T-cell mediated autoimmune pathogenesis [2]. It is frequently associated with other autoimmune diseases like autoimmune thyroiditis and vitiligo [1]. Lymphocytic infiltrates including both CD4+ and CD8+ T-cells exist in and around the hair follicles [1, 3]. T helper 1 (Th1) cytokines like IL-2 and IFN-*γ* play an important role in the disease process [2, 4]. The most popularly accepted mechanism for this disease is loss of immune privilege in the hair bulbs. Hair follicles are normally immune privileged and do not express major histocompatibility complex class II (MHC-II) molecules and also there is low expression of MHC-I molecules. Antigen presenting cells (APCs) are not present around normal hair bulbs. In AA, there is an increased expression of MHC-I and MHC-II molecules in the hair bulbs and APCs lie within the hair follicles in an increased number [3, 5]. There may be spontaneous hair regrowth, but most patients experience recurrence of the disease [1, 3]. No curative treatment is available and current therapies suppress activity of the disease [6, 7]. AA deteriorates quality of life and psychological status of the patients. Anxiety and depression are more common in AA patients than in the normal population [3, 5, 8].

The renin-angiotensin-aldosterone system (RAAS) is an endocrine axis that plays a role in blood pressure control. In this system, renin changes angiotensinogen to angiotensin I and angiotensin converting enzyme (ACE) changes angiotensin I to angiotensin II. Angiotensin II is the biologically active component of the system and acts via its receptors, especially type 1 receptor (AT1). RAAS blockers, like ACE inhibitors (ACEIs) and angiotensin receptor blockers (ARBs), are frequently used in the treatment of hypertensive disorders. Besides its major role, some studies have suggested a role for this system in inflammatory and autoimmune processes [9–11]. Based on this fact and with respect to the autoimmune nature of AA, the aim of our study was to evaluate the activity of RAAS in AA through measuring serum ACE activity in AA patients.

## 2. Methods

### 2.1. Patient Selection

In this case-control study, the case group consisted of clinically diagnosed patients with alopecia areata. These patients were selected from patients referring to Faghihi dermatology outpatient clinic, Shiraz University of Medical Sciences, southern Iran, between the years 2012 and 2013. Complete medical history was taken and physical examination (including blood pressure measurement) was performed for each case. Patients who could have altered function of the immune system or RAAS including those with history of hypertension, thyroid disorders, or other autoimmune diseases as well as those using topical or systemic immunosuppressive agents within the last two months were excluded from the study. Patients who had high blood pressure (more than 120/80 mmHg) at the time of visit were also excluded.

The control group included healthy subjects who were selected from individuals referring to Naderkazemi clinic, Shiraz University of Medical Sciences, for premarital tests. They were matched for age and sex with the case group.

### 2.2. Study Protocol and Assessments

Both groups were informed about the aim of the study and gave their informed consent. Information on patients' age, sex, disease duration, and severity was recorded. The patients were then divided into three groups based on disease severity, which was defined as follows [12]:

1. Mild: three or less than three patches of hair loss with maximum diameter of 3 cm.
2. Moderate: more than three patches of hair loss or at least a patch with a diameter above 3 cm.
3. Severe: alopecia totalis or universalis.

5 cc of blood was taken from each participant in this study. Blood samples were stored at room temperature for 20 minutes until clot was formed and then they were centrifuged for 10 minutes at 1500 ×g. Serum samples were collected and stored at −80°C until analyzed. ACE activity was assessed using a commercially available kit (Audit Diagnostics Company, Ireland) and an automated spectrophotometer (Mindray, BS 200, China) according to the procedure provided by the manufacturer. In this method, hydrolysis of substrate in the presence of serum ACE decreases absorbance at 340 nm, which is proportional to ACE activity.

### 2.3. Statistical Methods

According to a previous study which measured ACE activity in rheumatoid arthritis (RA) [11], median ACE levels were 48 and 33 in the patient and control groups, respectively. With common standard deviation of 15, power of 0.8, and type I error of 0.05, a minimum sample size of 16 was estimated for each group. The results were expressed as mean ± standard deviation. *T*-test was used to compare ACE activity between case and control groups. Pearson correlation coefficient was used to evaluate any correlation between ACE activity and disease duration and severity. Statistical package for the social sciences (SPSS) v.16.0 software was used to analyze the data. All results were considered significant if *P* values were 0.05 or less.

## 3. Results

Nineteen patients (63% male, 37% female) aged from 18 to 40 years old (mean age = 26.21 ± 5.16) were enrolled in our study as the case group. The control group consisted of 16 healthy subjects (56% male, 44% female) aged from 18 to 40 years old (mean age = 28.06 ± 5.44). Of the 19 patients, 9 (47.4%) had mild, 5 (26.3%) had moderate, and 5 (26.3%) had severe disease. Serum ACE activity was 17% higher in the case group than in the control group but the difference was not statistically significant (*P* = 0.085) (Table 1). There was no correlation between ACE activity and disease duration (*P* = 0.25) and severity (*P* = 0.13).

## 4. Discussion

Although the exact etiopathogenesis of AA is unknown, recent studies yield considerable evidence in support of a T-cell mediated autoimmune mechanism. Coexistence of AA with other autoimmune diseases [5, 13] and presence of autoimmune antibodies including autoantibodies against hair follicles [14, 15] have been reported. Rich inflammatory cell infiltrates consisting mainly of T-cells are present in and around hair bulbs [16, 17]. Several studies have suggested that cytokines may play an important role in the disease process. A Th1 cytokine profile with elevated levels of TNF-*α* [18], IL-1, IL-2 [4], and IFN-*γ* [2, 4] and low levels of TGF *β*1 due to improper function of regulatory T- (T reg) cells [19] has been reported in this disease. IFN-*γ* may play an important role in the pathogenesis of AA, and IFN-*γ* level may be an indicator of disease activity [2].

Besides its major role in the control of the arterial pressure, several studies suggest an important role for the RAAS in inflammatory and autoimmune processes. In a study by Sagawa et al. ARBs caused a decrease in IFN-*γ* production and lymphocyte proliferation and also improvement of the clinical and histopathologic features of arthritis in mice [20]. Shao et al. reported an increase in IFN-*γ* (a Th1 cytokine) and a decrease in IL-4 (a Th2 cytokine) following infusion of angiotensin II into mice. Administration of ARBs led to the improvement of this imbalance [21].

To the best of our knowledge, this is the first study to evaluate the role of RAAS in AA as an autoimmune disease. There are some experiments on other autoimmune diseases like RA and multiple sclerosis (MS) where Th1 cytokines play a key role. A study on MS by Platten et al. showed increased activity of the RAAS. AT1 receptor was upregulated in CD4+ T-cells involved in the autoimmune process. Blocking the RAAS by ACEIs and ARBs suppressed autoreacitve Th1 and Th17 cells and induced T reg cells [9]. In studies by Çobankara et al. and Veale et al., serum ACE levels were higher in RA patients than in the control group but no statistically significant difference was seen. On the other hand, ACE activity was significantly higher in synovial fluids of the patients [11, 22]. Similarly, in our study, mean serum ACE activity was higher (17%) in AA patients than in controls but no statistically significant difference was observed. This increase in serum ACE activity may indicate the local involvement of RAAS in the pathogenesis of AA.

The RAAS is traditionally known to function in blood circulation but recent studies provide evidence on the existence of this system in tissues [10]. Platten et al. confirmed the locally increased activity of the RAAS in brain lesions of MS [9]. Goto et al. obtained peripheral blood monocytes of RA patients and observed that, in a serum-free condition, these monocytes spontaneously produced and released increased amounts of IL-1 and ACE [23]. Çobankara et al. found that ACE levels were significantly higher in synovial fluids of RA patients. They concluded that increased local production of ACE led to joint destruction in this disease [11]. Veale et al. also found that ACE levels were significantly higher in synovial fluids of RA patients. They used anti ACE monoclonal antibodies to stain synovial membrane samples of RA patients and localized ACE to endothelial cells and mononuclear cells of macrophage origin. They concluded that ACE is produced by the synovial membrane in RA [22]. Due to some limitations in the present study, it was not feasible to measure local and serum ACE activity concomitantly. The increased serum ACE activity found in this study, although statistically insignificant, may indicate locally increased activity of the RAAS, since the soluble form of ACE may be an indicator of turnover and clearance of the membrane-bound form [10]. Therefore, our findings suggest that the RAAS may be involved in the pathogenesis of AA. However, assessment of ACE activity in tissue samples of AA patients seems to be a more sensitive indicator of the activity of this system in this condition.

## 5. Conclusion

The increased serum ACE activity found in the present study may show local involvement of the RAAS in the pathogenesis of AA. Assessment of ACE in studies with a larger sample size and also in tissue samples can help to evaluate the role of RAAS in AA. This may provide a rationale to introduce RAAS blockers, which are inexpensive and relatively safe, as a new choice for the treatment of this disease.

## Figures and Tables

**Table 1:** ACE activity in patient and control groups [expressed as mean ± standard deviation].

Parameter	Patient group (*n* = 19)	Control group (*n* = 16)	*P* value
Serum ACE activity (U/L)	55.81 ± 16.03	46.41 ± 15.08	0.085
